# Effects of (*R*)- and (*S*)-α-Hydroxylation of Acyl Chains in Sphingosine, Dihydrosphingosine, and Phytosphingosine Ceramides on Phase Behavior and Permeability of Skin Lipid Models

**DOI:** 10.3390/ijms22147468

**Published:** 2021-07-12

**Authors:** Andrej Kováčik, Petra Pullmannová, Lukáš Opálka, Michaela Šilarová, Jaroslav Maixner, Kateřina Vávrová

**Affiliations:** 1 Skin Barrier Research Group, Faculty of Pharmacy in Hradec Králové, Charles University, Akademika Heyrovského 1203, 500 05 Hradec Králové, Czech Republic; pullmanp@faf.cuni.cz (P.P.); OPALL6AA@faf.cuni.cz (L.O.); silaromi1@gmail.com (M.Š.); vavrovak@faf.cuni.cz (K.V.); 2 Faculty of Chemical Technology, University of Chemistry and Technology in Prague, Technická 5, 166 28 Prague, Czech Republic; maixnerj@vscht.cz

**Keywords:** skin barrier, stratum corneum, lipids, ceramides, hydroxylation, biophysics, permeability

## Abstract

Ceramides (Cers) with α-hydroxylated acyl chains comprise about a third of all extractable skin Cers and are required for permeability barrier homeostasis. We have probed here the effects of Cer hydroxylation on their behavior in lipid models comprising the major SC lipids, Cer/free fatty acids (C _16_-C _24_)/cholesterol, and a minor component, cholesteryl sulfate. Namely, Cers with (*R*)-α-hydroxy lignoceroyl chains attached to sphingosine (Cer AS), dihydrosphingosine (Cer AdS), and phytosphingosine (Cer AP) were compared to their unnatural (*S*)-diastereomers and to Cers with non-hydroxylated lignoceroyl chains attached to sphingosine (Cer NS), dihydrosphingosine (Cer NdS), and phytosphingosine (Cer NP). By comparing several biophysical parameters (lamellar organization by X-ray diffraction, chain order, lateral packing, phase transitions, and lipid mixing by infrared spectroscopy using deuterated lipids) and the permeabilities of these models (water loss and two permeability markers), we conclude that there is no general or common consequence of Cer α-hydroxylation. Instead, we found a rich mix of effects, highly dependent on the sphingoid base chain, configuration at the α-carbon, and permeability marker used. We found that the model membranes with unnatural Cer (*S*)-AS have fewer orthorhombically packed lipid chains than those based on the (*R*)-diastereomer. In addition, physiological (*R*)-configuration decreases the permeability of membranes, with Cer (*R*)-AdS to theophylline, and increases the lipid chain order in model systems with natural Cer (*R*)-AP. Thus, each Cer subclass makes a distinct contribution to the structural organization and function of the skin lipid barrier.

## 1. Introduction

The primary function of mammalian skin is to provide a semipermeable interface between the body and the external environment, i.e., prevent water loss and the penetration of exogenous, potentially harmful compounds [1]. The permeability barrier to most substances is localized in the uppermost skin layer, the *stratum corneum* (SC), particularly in its extracellular lipid matrix. The composition and organization of these SC lipids are somewhat unusual: the dominant lipids comprise ceramides (Cers), cholesterol (Chol), and free fatty acids (FFAs), which form multiple lamellae of tightly packed lipids with mostly rigid chains [2,3,4,5]. The SC Cers consist of a sphingoid base (sphingosine (S), dihydrosphingosine (dS), phytosphingosine (P), and 6-hydroxysphingosine (H)) amide-linked to fatty acid acyl (which is non-substituted (N), α-hydroxylated (A), ω-hydroxylated (O), or bears the ω-linoleyloxy group (EO)). The shorthand nomenclature [6] uses the combination of the letters in parentheses; for example, *N*-α-hydroxyacyl sphingosine is Cer AS.

The behavior of skin-identical Cers with very long non-hydroxylated acyl chains has been widely explored in model biological membranes. The absence of a *trans*-double bond in Cer precursors, dihydroceramides, that form approximately 12% of Cers in healthy SC [7] did not affect the permeability of skin lipid models [8]. The shortening of the acyl or sphingoid chain in canonical sphingosine-based Cers decreased their barrier properties [9,10]. Cer NS with a very long acyl chain (24 carbons) seems essential for forming tightly packed impermeable lipid lamellae in the SC [11]. In comparison, the shortening of the acyl chain by eight carbons directly contributes to a less effective lipid barrier, similar to the defects described in atopic skin [12]. Biophysical studies have suggested that phytoceramides, formed by dihydroceramide desaturase 2 [13], can probably reduce structural lipid defects in the SC lipid matrix by the formation of hydrogen bonding [14,15]. In contrast, permeability studies have revealed that Cer NP leads to a less effective barrier than Cer NS in model SC lipid systems [16]. Cer NH, a unique epidermal-specific Cer subclass with unknown biosynthesis, contributes to forming an atypical long lamellar phase in SC lipids [17].

Cers with α-hydroxylated acyl chains (AS, AdS, AP, and AH) comprise about a third of all extractable skin Cers [18] and are required for permeability barrier homeostasis [19]. The synthesis of such Cers requires fatty acid 2-hydroxylase [19], but there is also another, yet unidentified, enzyme (phytanoylCoA 2-hydroxylase is a potential candidate) [20,21]. This hydroxylation is stereospecific as only (*R*)-isomers (i.e., D-isomers) have been found in mammalian sphingolipids [22].

Skin α-hydroxylated Cers have been investigated—either alone [23,24] or in model SC lipid systems [25,26,27,28,29]—by differential scanning calorimetry, infrared spectroscopy, NMR spectroscopy, and diffraction methods. However, most studies have been limited to Cers with 2-hydroxyoctadecanoyl chains [25,26,27], which are considerably shorter than SC Cer acyl chains and make the molecules symmetrical, affecting their behavior and barrier properties [11,12]. Few researchers have probed the behavior of Cers with α-hydroxylated very long acyl tails (e.g*.,* 24 carbons) [29]. The relationships between Cer hydroxylation patterns (including their stereochemistry) and their ability to restrict water loss and permeability are not well understood.

**Figure 1:**
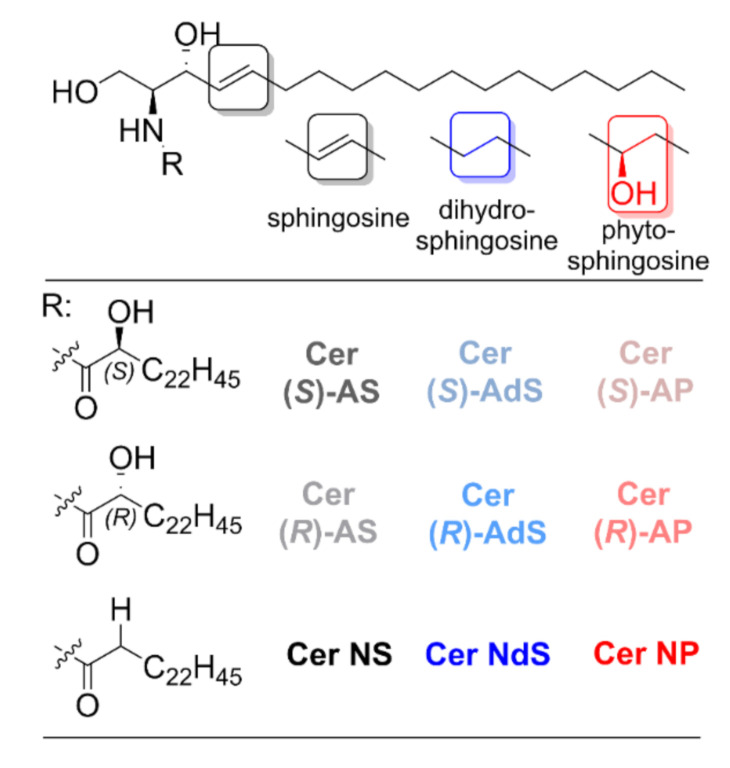
Structures of the α-hydroxylated Cers with physiological (i.e*.,* Cer (*R*)-AS, Cer (*R*)-AdS, and Cer (*R*)-AP) and unnatural stereochemistry (i.e*.,* Cer (*S*)-AS, Cer (*S*)-AdS, and Cer (*S*)-AP) and their non-hydroxylated counterparts (i.e*.,* Cer NS, Cer NdS, and Cer NP) investigated in this work.

We aim at probing the effects of α-hydroxylation in three Cer subclasses (AS, AdS, and AP, all with 24 carbon acyl chains) on the lipid organization and permeability of SC lipid model systems composed of Cer, Chol, FFAs (16–24 carbons), and cholesteryl sulfate (CholS). The α-hydroxy Cers were compared to their non-hydroxylated counterparts (Cer NS, NdS, and NP). Furthermore, we compared the Cers with α-hydroxyls in natural (*R*) and unnatural (*S*) configurations (Figure 1). All Cers had 24 carbon acyl chains. The detailed lipid composition of the investigated lipid mixtures is shown in Table 1. The lamellar lipid arrangement is described using X-ray diffraction (XRD). Lipid chain order, lateral packing, mixing, and thermotropic behavior of the model systems were investigated by Fourier transform infrared spectroscopy (FTIR) using deuterated lipids. The permeability of the SC lipid films was probed using three markers (water loss, theophylline (TH), and indomethacin (IND)).

## 2. Results

### 2.1. Lamellar Lipid Arrangement

**Figure 2:**
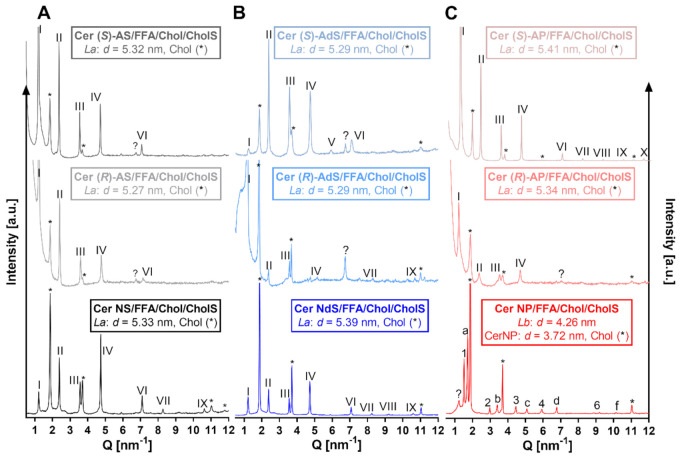
XRD diffractograms of Cer/FFAs/Chol/CholS membranes containing either sphingosine (Cer NS, Cer (*R*)–AS, or Cer (*S*)–AS; Panel (**A**) or dihydrosphingosine (Cer NdS, Cer (*R*)–AdS, or Cer (*S*)–AdS; Panel (**B**) or phytosphingosine Cers (Cer NP, Cer (*R*)-AP, or Cer (*S*)–AP; Panel (**C**) at ambient room humidity. Intensity is given in arbitrary units (a.u.). Roman numerals mark the *La* phase (*d* = 5.3–5.6 nm); Arabic numerals mark the *Lb* phase (*d* = 4.3 nm); letters belong to the phase of crystalline Cer NP (*d* = 3.7 nm); asterisks mark separated Chol (*d* = 3.4 nm).

First, we studied the microstructure of our lipid models using XRD. All samples, except those with Cer NP, contained a set of diffraction peaks that provided a lamellar phase, *La,* with a repeat distance, *d* = 5.3–5.4 nm (marked by Roman numerals; Figure 2). First-order peaks in all *La* lamellar phases have lower relative intensity, which is caused by technical reasons because of a divergence slit programmed for constant irradiated length in performed measurements; thus, the intensity and depth of the incident beam will vary. The differences in *d* between replicates were less than 0.01 nm unless otherwise indicated. This repeat distance is close to the so-called short periodicity phase (*d* = 5.3– 6.5 nm) found in model skin lipid membranes [9,16,30] and human SC [31,32]. In all studied membranes, separated Chol (or its mixture with CholS) was also found (reflections marked by asterisks; repeat distance, *d* = 3.4 nm; Figure 2) [9,32,33]. The Cer NP-based sample revealed a phase separation similar to previous findings [17]. The peaks were assigned to the phase of separated Cer NP (*d* = 3.72 nm) [34,35], Chol, an L*b* phase with *d* = 4.26 nm, and two weak peaks that correspond to lignoceric acid. Thus, α-hydroxylation in the Cer acyl chain, either at natural (*R*) or unnatural (*S*) stereochemistry, did not dramatically change the lamellar repeat distances in our model skin lipid mixtures. The exceptions were phytosphingosine-based Cer AP, either (*R*) or (*S*) isomers, where α-hydroxylation improved lipid miscibility over the Cer NP system.

### 2.2. Lipid Chain Order

**Figure 3:**
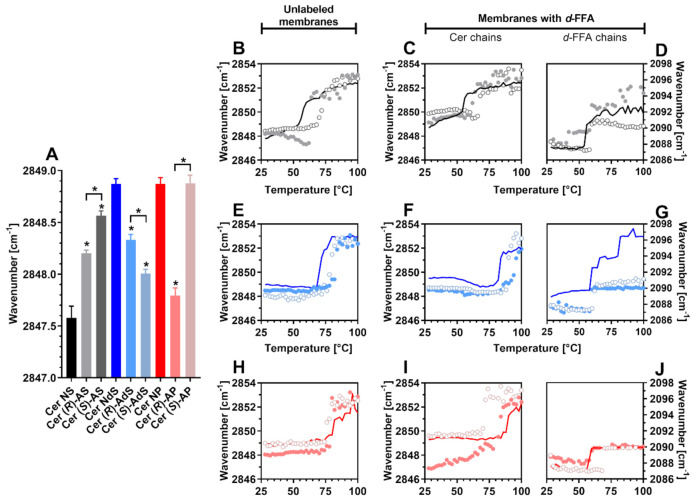
Chain order and phase transitions (FTIR spectra) in Cer/(*d*)-FFAs/Chol/CholS membranes containing either sphingosine (Cer NS, Cer (*R*)-AS, or Cer (*S*)-AS; Panels (**B**–**D**) or dihydrosphingosine (Cer NdS, Cer (*R*)-AdS, or Cer (*S*)-AdS; Panels (**E**–**G**) or phytosphingosine Cers (Cer NP, Cer (*R*)-AP, or Cer (*S*)-AP; Panels (**H**–**J**) at ambient room humidity. Panel (**A**) shows methylene symmetric stretching wavenumbers at a physiological temperature of 32 °C as means ± SEM, *n* = 4. Asterisks indicate statistically significant differences against the controls with non-hydroxylated Cers (Cer NS or Cer NdS or Cer NP) (*p* < 0.05). The graphs in panels (**B**,**E**,**H**) show the temperature dependence of the methylene symmetric stretching FTIR spectra of model SC lipid membranes with unlabeled lipids. The lines belong to membranes based on non-hydroxylated Cers, filled circles belong to membranes based on (*R*)-α-hydroxy Cers, and open circles belong to (*S*)-α-hydroxy Cer membranes. The behavior of the samples with *d*-FFAs is shown in the panels: (**C**,**D**) sphingosine Cers, (**F**,**G**) dihydrosphingosine Cers, and (**I**,**J**) phytosphingosine Cers, where Panels (**C**,**F**,**I**) describe the Cer chains (CH_2_ vibrations) and Panels (**D,G**,**J**) show the *d*-FFA chains (CD_2_ vibrations).

Next, we studied the lipid systems using FTIR. At skin temperature (32 °C), the lipid chains in all membranes were well ordered, with relatively high proportions of all-*trans* conformers (indicated by methylene symmetric stretching wavenumbers below 2850 cm^−1^) [36] (Figure 3A). The Cer NS models had the most ordered lipids (2847.6 ± 0.1 cm^−1^ at 32 °C) of all the studied systems. The effects of α-hydroxylation of Cer acyl were highly dependent on the sphingoid base: Cer AS isomers gave less ordered structures than Cer NS (by up to 1 cm^−1^), whereas Cer AdS mixtures were more ordered than the sample with Cer NdS. The differences in chain order between the natural and unnatural isomers were minor (albeit reproducible and statistically significant). Notably, the chain order in the model with unnatural Cer (*S*)-AP was similar to Cer NP and by over 1 cm^−1^ lower than for Cer (*R*)-AP membranes (Figure 3A). Note that these differences may result from a mixture of effects as the Cer NS lipid models undergo an orthorhombic to hexagonal transition, which starts below 32 °C and ends at 37 °C, while the others do not.

### 2.3. Phase Transitions

Lipid phase transitions can be deduced from the shift of methylene stretching to higher wavenumbers in accordance with temperature [36,37] (Figure 3B–J). The melting points of the studied Cers (Figure S1) suggest that saturation and hydroxylation of the sphingosine chain increase the energy required for chain disordering. The effects of α-hydroxylation of the acyl chain depend on the stereochemistry of the hydroxyl (natural (*R*) isomers disorder at lower temperatures than (*S*) ones) and sphingoid base (both Cer AP isomers with 4 hydroxyls had lower melting points than Cer NP isomers with 3 hydroxyls).

In the Cer NS/FFAs/Chol/CholS mixture, the chains disordered at 57 °C, in agreement with previous studies [9,11]. In that sample, both Cer (CH_2_) and *d*-FFAs (CD_2_) underwent transitions at similar temperatures, suggesting their good miscibility (Table 2). The stretching wavenumbers in the Cer (*R*)-AS/FFAs/Chol/CholS models decreased at approximately 50 °C, suggesting lipid reorganization (likely involving some FFA chains, as d-FFA stretching shifted to higher wavenumbers at this temperature), and melted at 63 °C (Figure 3B). The individual components, (*R*)-Cer AS and *d*-FFAs, disordered at around 60 °C (Figure 3C,D). With unnatural (*S*)-Cer AS, phase transition occurred at a similar temperature as with (*R*)-Cer AS but without any prior chain reordering (Figure 3B). The *d*-FFA and (*S*)-Cer AS chain melting transitions were separated (Figure 3C,D), indicating their limited mixing.

Lipid mixtures with Cer NdS disordered at a higher temperature than those with Cer NS, consistent with the higher melting point of Cer NdS over Cer NS and previous results describing the formation of more thermally stable ordered domains of Cer NdS than Cer NS [8,17,38]. α-Hydroxylation of Cer NdS increased the lipid chain order at low temperatures and increased the main transition temperatures without any marked differences between (*R*) and (*S*) isomers (Figure 3E–G). The Cer and *d*-FFA behavior indicated their limited mixing.

The thermotropic behavior of mixtures with phytoceramides NP and AP is depicted in Figure 3H–J. The Cer NP-based lipid model disordered at a higher temperature than the model with Cer NdS, consistent with the higher melting point of Cer NP over Cer NdS and previous data [16]. The phase separation seen by XRD was confirmed by markedly different disordering temperatures of Cer NP and *d*-FFAs in that sample. The α-hydroxylation of Cer NP (increasing the hydroxyl count to 4) did not further increase but slightly decreased the overall chain disordering temperature. The natural (*R*)-Cer AP chains were more ordered than those of (*S*)-Cer AP, and the gaps between the Cer AP and *d*-FFA melting temperatures were narrower than between Cer NP and *d*-FFAs (Table 2).

### 2.4. Lateral Chain Packing and Lipid Mixing

**Figure 4:**
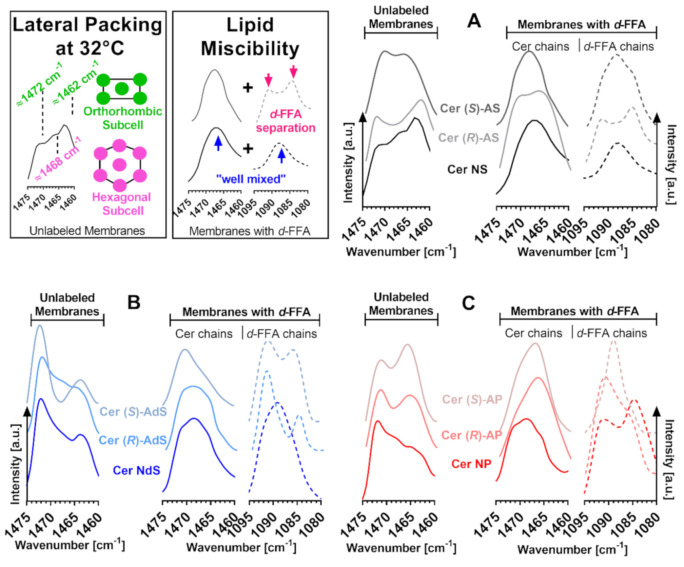
FTIR spectra (methylene scissoring vibrations) of model SC lipid membranes containing the studied Cers: sphingosine Cers (Cer NS, Cer (*R*)–AS, or Cer (*S*)–AS; Panel (**A**), dihydrosphingosine Cers (Cer NdS, Cer (*R*)–AdS, or Cer (*S*)–AdS; Panel (**B**), phytosphingosine Cers (Cer NP, Cer (*R*)–AP, or Cer (*S*)–AP; Panel (**C**), (*d*)–FFAs, Chol, and CholS. The first graphs in each panel show the behavior of the membrane with unlabeled lipids, i.e., both Cer and FFA chains. The second and third graphs in each panel show the behavior of the membrane with *d*-FFAs, where the second graph shows the CH_2_ vibrations of Cer chains, and the third graph shows the CD_2_ vibrations of *d*-FFAs. The splitting of the scissoring band into a doublet is indicative of orthorhombic chain packing. The thermal evolution of the prominent bands is given in the supporting information (Figure S3).

The infrared methylene scissoring and rocking bands are sensitive to lateral chain packing. In all studied model membranes, both scissoring (Figure 4, wavenumbers ~1472 and ~1462 cm^−1^) and rocking bands (~730 and ~719 cm^−1^; Figure S2) were split into doublets at 32 °C, indicating tight orthorhombic chain packing. With increasing temperature, the orthorhombic doublet either merges into a hexagonal singlet before the chain disordering (e.g*.,* in the Cer NS membrane) or collapses with the main phase transition (e.g*.,* in (*S*)-Cer AdS; Figure S3). The relative proportions of orthorhombic packing based on the intensity ratios of the 730 and ~719 cm^−1^ components of the rocking bands are given in Figure S2. These relative proportions are not quantitative because the extinction coefficients for the orthorhombic vs. hexagonal phases are unknown. However, this simple comparison suggests that whereas the α-hydroxylation of Cer NS decreases the proportion of orthorhombically packed chains, the α-hydroxylation of Cer NdS and Cer NP has the opposite effect. In addition, the content of orthorhombic lipids in the Cer NS sample is likely lowered by the already occurring orthorhombic–hexagonal transition in this temperature region. This transition has a negligible effect on the orthorhombic phase content in all other samples as this transition occurs at ≥60 °C.

Lipid chain deuteration shifts the methylene scissoring wavenumbers to approximately 1090 cm^−1^ and enables the examination of the CH_2_ and CD_2_ chains individually. When the CH_2_ scissoring doublet changes into CH_2_ and CD_2_ singlets (wavenumbers ~1468 and ~1088 cm^−1^) upon deuteration of one component of the lipid mixture, it indicates the mixing of the protonated (unlabeled) and deuterated (labeled) components because the vibrational coupling does not occur between different isotopes. Such behavior, indicative of good Cer-FFA mixing, was observed in the Cer NS and (partly) Cer NdS samples, whereas *d*-FFAs were clearly separated in the Cer NP sample, consistent with previous results [16,17] (Figure 4). In general, the α-hydroxylation in Cer AS and AdS compromised their mixing with FFAs compared to non-hydroxylated Cer NS or Cer NdS, whereas the α-hydroxylation of Cer NP improved the affinity of the resulting Cer AP to FFAs. These effects were further modulated by the α-hydroxyl’s stereochemistry (Figure 4).

### 2.5. Model Membrane Permeability

**Figure 5:**
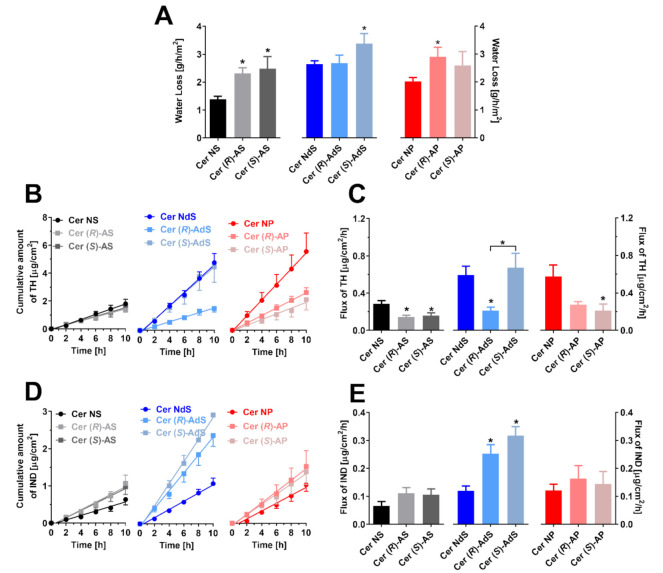
The permeabilities of the lipid films containing the studied Cers, FFAs, Chol, and CholS: water loss (Panel **A**), TH permeation profiles (**B**) and TH flux values (**C**), IND permeation profiles (**D**), and IND flux values (**E**). Means ± SEM, *n* > 6. The asterisks indicate significant differences against respective non-hydroxylated Cers at *p* < 0.05.

To elucidate the effects of Cer α-hydroxylation and stereochemistry on the barrier properties of the studied models, we placed the lipid films on porous support in Franz diffusion cells and measured water loss and two permeation markers. Water loss through the control Cer NS samples was 1.67 ± 0.13 g/h/m^2^ (Figure 5A), consistent with previous studies [8,9,17]. Saturation of the sphingosine double bond in Cer NdS and Cer NP increased water loss (2.65 ± 0.12 and 2.02 ± 0.14 g/h/m^2^, respectively). α-Hydroxylation of Cers in these models led to approximately 30–50% higher water loss, except for Cer (*R*)-AdS, where this parameter did not change over to Cer NdS.

In contrast, the permeability of the lipid films to TH, a small molecule with balanced lipophilicity (that is expected to cross the membrane via free-volume diffusion), was decreased upon Cer α-hydroxylation (with a single exception, Cer (*S*)-AdS). Notably, a significant difference was found between samples with Cer (*R*)-AdS and Cer (*S*)-AdS diastereomers (Figure 5B,C). The third marker, larger and more lipophilic IND (that would likely permeate by lateral diffusion), revealed other results. The resistance of the lipid mixtures to this molecule was unchanged upon α-hydroxylation, except for both Cer AdS isomers, which gave approximately twice higher permeability than the parent Cer NdS (Figure 5D,E).

## 3. Discussion

The major forces that stabilize lipid membranes arise from the lateral interactions of lipid hydrophobic chains. One way of increasing the stability of the lipid assemblies further is by adding hydrogen bonding groups such as hydroxyl groups. Theoretically, such a hydrogen-bonding network could promote close lipid packing and improve the barrier for polar and lipophilic compounds [39]. To probe the effects of α-hydroxylation on SC Cers, we compared Cers with α-hydroxylated acyl chains (Cer AS, AdS, AP, either (*R*) or (*S*) diastereomers at α-carbon) with their non-hydroxylated counterparts, Cer NS, NdS, and NP.

Cer AS comprises approximately 4–10 molar% [18,40] of all SC Cers. Compared to its non-hydroxylated counterpart, Cer NS (a canonical, most widely studied Cer [9,10,11,12,17,41]), α-hydroxylation in Cer AS did not change the lamellar repeat distance found in the Cer/FFAs/Chol/CholS model but decreased the overall lipid chain order, which led to some phase separation (in agreement with Garidel et al. [25]). The model with unnatural (*S*)-Cer AS also had less orthorhombically packed lipid chains. Consistent with the worse chain order and lipid mixing, the resistance of the model to water loss decreased. However, the permeability of the lipid film to TH and IND decreased or remained unchanged, respectively, showing that permeability to water and various substances is sensitive to different biophysical parameters. The lack of marked differences among the pair of Cer diastereomers, (*R*)-AS and (*S*)-AS, was somewhat unexpected as previous studies have described an intramolecular hydrogen bond in (*S*)-AS that affects its interfacial behavior [39]. Moreover, the conversion of C−3 stereochemistry of the sphingosine chain in Cer NS changed the Cer’s mixing with other SC lipids and increased the model’s permeability [38].

Cer AS is an isomer of another human SC Cer species, Cer NH. They both have a C-4/5 *trans*-double bond in the sphingoid base and an additional hydroxyl either at C-6 in Cer NH or the α-position of the acyl chain in Cer AS. Notably, these Cers often coelute in the TLC analysis of SC lipids. A previous study showed that Cer NH [17] led to FFA separation, and the models had a similar chain order and transition temperatures like those with Cer AS. However, the models with Cer NH formed a phase with 10.6 nm periodicity (besides *La*), which was not observed here with either Cer AS diastereomer.

Natural precursors of Cers, dihydroceramides, are less abundant and less studied. The lack of C4–5 desaturation in Cer NdS disordered the lipids, loosened their packing, and increased the model’s permeability compared to Cer NS, in agreement with published studies [8,16,38]. α-Hydroxylation in Cer AdS did not affect the lamellar repeat distance, increased chain order and packing, but decreased Cer mixing with FFAs (compared to its non-hydroxylated counterpart Cer NdS). Consequently, the model’s permeability was mainly unchanged or increased, with one exception: the permeability of the model with (*R*)-Cer AdS to TH was significantly lower compared to the (*S*)-Cer AdS model.

Notably, Cer AdS and Cer NP are isomers that differ only in the position of one hydroxyl, which is in the acyl chain or sphingoid base chain, respectively. Cer NP is the most abundant and widely investigated [24,25,42,43] SC Cer (approximately 23 molar%) [18]. Like Cer AdS, Cer NP does not mix well with FFAs, and the lipids disorder at similar temperatures. Nevertheless, XRD of the Cer NP model showed the separation of a 3.7 nm phase that most likely corresponds to a separated V-shaped Cer NP [42], whereas the Cer AS systems formed a *La* phase (consistent with Schmitt et al. [29]), which likely corresponds to the SC short periodicity phase. Notably, Cer NP models had similar permeabilities to Cer NdS models, indicating that hydroxylation of the dihydrosphingosine chain at C-4 has less impact on the model’s barrier properties than α-hydroxylation. However, according to Uche et al., the phytoceramide-based membranes (composed of Cer EOS/Cer NP/FFAs/Chol, 0.4:0.6:1:1 mol) are less permeable to *p*-aminobenzoic acid than their sphingosine counterparts (composed of Cer EOS/Cer NS/FFAs/Chol, 0.4:0.6:1:1 mol) [44]. Thus, the difference between Cers also depends on model composition and the model permeant used.

Cer AP with four hydroxyl groups is the second most polar SC Cer (after Cer AH), and it comprises 9–14 molar% [18,40] of SC Cer. The physiological Cer (*R*)-AP led to the formation of the *La* lamellar phase, corresponding to the short periodicity phase, consistent with a study that also used Cer (*R*)-AP with a 24-carbon acyl chain [29]. This phase was also found in a model where the Cer fraction comprised a Cer AP/NS 1:1 molar mixture [45]. In contrast to Schmitt et al., we also observed a phase with a 5.4 nm repeat distance for the unnatural (*S*)-AP isomer. This difference is probably due to different membrane compositions (Schmitt et al. investigated membranes containing Cer AP/lignoceric acid/Chol in a 1:1:0.7 molar ratio). Raudenkolb et al. also described differences between Cer AP diastereoisomers with 18-carbon acyl chains [23]. The only significant difference between Cer AP diastereomers we saw here was in lipid chain order, which was lower in the unnatural isomer. This discrepancy is likely connected with the slightly different model compositions and different lipid treatments between our model and the published models as the SC lipids are highly polymorphic [46]. In general, α-hydroxylation in Cer AP improved lipid miscibility and lateral packing in our models compared to Cer NP. Our barrier function markers again responded differently to this structural change; whereas water loss increased, permeability to TH was decreased, and IND flux did not change.

Although there are several studies that have elucidated many of the functions of Cer in SC, Cer structural diversity is still fascinating. It is not clear why the skin creates additional hydroxyl groups in several barrier Cers, which were designed as hydrophobic components of the skin barrier to prevent water loss. The difference in the lipid polar head group (double bond, hydroxyl group in the sphingoid base/acyl chain) might affect lateral and lamellar lipid organization and the formation of hydrogen bonding. These simple structural variations affect skin barrier function in various mammalian species. For example, phytosphingosine Cers (mostly Cer NP and Cer AP) are more abundant than sphingosine Cers (Cer NS and Cer AS) in humans; the sphingosine Cers prevail in porcine SC. In addition, Cer AS is considerably shorter (mostly C _16_ acyl chains) in porcine skin [47,48,49,50]. Our data suggest that Cer hydroxylation and stereochemistry affect their ability to maintain the skin lipid barrier differently. This is essential knowledge for computer modeling of simple and also complex lipid mixtures. These results would serve as a starting point to probe the behavior of such Cers (e.g*.,* deuterated) in multicomponent mixtures. Our results should also be considered in future studies using similar lipid mixtures based on a single Cer as models of the skin lipid barrier for studying the interaction of various compounds such as permeation enhancers as such interaction would likely differ in Cer NS and Cer NP models.

In conclusion, we have probed here the effects of Cer hydroxylation on their behavior in lipid models comprising the major SC lipids, Cer/FFAs/Chol, and a minor component, CholS. Namely, Cers with α-hydroxylated acyl chains (Cer AS, AdS, AP) with the hydroxyl in a natural (*R*) configuration were compared to their unnatural (*S*) diastereomers and to their non-hydroxylated counterparts, Cer NS, NdS, and NP. By comparing several biophysical parameters and the permeabilities of these models, we conclude that there is no general or common consequence of Cer α-hydroxylation. Instead, we found a rich mix of effects, highly dependent on the sphingoid base chain, configuration at the α-carbon, and permeability marker used. Thus, each Cer subclass makes a distinct contribution to the structural organization and barrier function of the SC.

## 4. Materials and Methods

### 4.1. Chemicals

Cer (*R*)-AdS, Cer (*S*)-AdS, and Cer (*S*)-AP were synthesized according to the procedure described for Cer AP [45]. Cer NS (synthetic, over 99% stereochemically pure, i.e., (2*S*,3*R*,4*E*)), Cer NdS (synthetic, over 99% stereochemically pure, i.e., (2*S*,3*R*)), Cer NP (synthetic, over 99% stereochemically pure, i.e., (2*S*,3*S*,4*R*)), Cer (*R*)-AS (synthetic, over 99% stereochemically pure, i.e., (2*S*,3*R*,2′*R*)), Cer (*S*)-AS (synthetic, over 99% stereochemically pure, i.e., (2*S*,3*R*,2′*S*)), and Cer (*R*)-AP (synthetic, over 99% stereochemically pure, i.e., (2*S*,3*S*,4*R*,2′*R*)) were purchased from Avanti Polar Lipids (Alabaster, AL, USA). Perdeuterated FFAs (*d*-FFAs, over 98.9% D) were purchased from CDN Isotopes (Pointe-Claire, Canada). All other chemicals, including Chol, CholS, and unlabeled FFAs, were purchased from Sigma-Aldrich (Schnelldorf, Germany). Water was deionized, distilled, and filtered through a Millipore Q purification system.

### 4.2. Preparation of the Lipid Membranes

The model SC lipid membranes were prepared as equimolar mixtures of Cer, Chol, and FFAs, with the addition of 5 wt% of CholS, as described previously [17]. FFAs (or *d*-FFAs) were mixed in a molar percentage corresponding to the native composition of human skin FFAs [51]. The lipids were dissolved in hexane/96% EtOH (2:1, *v*/*v*) at 4.5 mg/mL (note: the use of 96% aq. EtOH is necessary to dissolve CholS). These lipid solutions (3 × 100 µL per cm^2^) were slowly sprayed on Nuclepore polycarbonate filters with 15 nm pores (Whatman, Kent, UK) under a stream of nitrogen using a Linomat V (Camag, Muttenz, Switzerland) equipped with additional *y*-axis movement. These lipid films were heated to 90 °C, equilibrated for 10 min, and then slowly (~3 h) cooled to room temperature. Then, the membranes were equilibrated at 32 °C and 30% air humidity for 24 h. The lipid films for FTIR experiments were prepared in the same way. The homogeneity of the membranes was previously validated [12].

### 4.3. X-ray Diffraction (XRD)

The lipid mixtures for XRD measurements were prepared in the same manner as those for the permeation experiment. The lipids were sprayed onto a 22 × 22 mm cover glass instead of polycarbonate filters. The XRD data were collected at ambient temperature on an X’Pert PRO θ-θ powder diffractometer (PANalytical B.V., Almelo, The Netherlands) with parafocusing Bragg-Brentano geometry using CuKα radiation (λ = 1.5418 Å, U = 40 kV, I = 30 mA) in modified sample holders over the angular range of 0.6–30° (2θ). Data were scanned with an ultrafast position-sensitive linear (1D) detector X’Celerator with a step size of 0.0167° (2θ) and a counting time of 20.32 s/step [17].

### 4.4. Attenuated Total Reflectance Fourier Transform Infrared Spectroscopy (FTIR)

Infrared spectra of the model SC lipid membranes were collected on a Nicolet 6700 spectrometer (Thermo Scientific, Waltham, MA, USA) equipped with a single-reflection MIRacle ATR ZnSe crystal (PIKE technologies, Madison, WI, USA). A clamping mechanism with constant pressure was used. The spectra were generated by the co-addition of 256 scans collected at a resolution of 2 cm^−1^. The temperature dependence of the FTIR spectra was studied over the range of 28–100 °C with 2 °C steps using a temperature control module (PIKE Technologies, Madison, WI, USA). After each temperature increment, the sample was allowed to stabilize for 6 min before the spectrum was measured. The spectra were analyzed using Bruker OPUS software. The exact peak positions were determined from the second derivative spectra [17].

### 4.5. Water Loss through the Lipid Membranes

Water loss through the model membranes [g/h/m^2^] was measured using a Tewameter^®^ TM 300 probe and a Multi Probe Adapter Cutometer^®^ MPA 580 (CK electronic GmbH, Köln, Germany). The environmental conditions were comparable during all measurements: ambient air temperature of 25–26 °C and relative air humidity of 38–43%. The whole procedure was validated [12].

### 4.6. Permeation Experiments

The lipid membranes were mounted onto Franz diffusion cells with an available diffusion area of 0.5 cm^2^, with the lipid film facing the donor compartment. The acceptor compartment of the cell (6.5 ± 0.3 mL) was filled with phosphate-buffered saline (PBS, containing 10 mM phosphate buffer, 137 mM NaCl, and 2.7 mM KCl) at pH 7.4 with 50 mg/L gentamicin. The acceptor phase was stirred at 32 °C throughout the experiment. After 12-h equilibration, 100 µL of the model permeant—either 5% theophylline (TH) or 2% indomethacin (IND) suspensions in 60% propylene glycol—was applied into the membrane. Propylene glycol had no adverse effects on the membranes [9,12,16]. Samples of the acceptor phase (300 µL) were withdrawn every 2 h over 10 h to reach a steady-state situation and were replaced with the same volume of PBS. TH and IND were analyzed by validated HPLC methods [12,17].

### 4.7. Data Analysis

The cumulative amount of the drug that penetrated the lipid membrane (corrected for acceptor phase replacement; (μg/cm^2^)) was plotted against time [h], and the steady-state flux [μg/h/cm^2^] was calculated from the linear region of the plot. Data are presented as the mean ± standard error of the mean (SEM), and the number of replicates is given in the pertinent figure. One-way analysis of variance (ANOVA) with Dunnettʹs post hoc test method was used for statistical analysis, and *p* < 0.05 was considered significant.

## Figures and Tables

**Table 1:** The composition of model membranes used in this study.

Membrane Name	Model Membrane Composition
Cer NS	Cer NS/FFAs/Chol (1:1:1 mol) + 5 wt% of CholS
Cer (*R*)-AS	Cer (*R*)-AS/FFAs/Chol (1:1:1 mol) + 5 wt% of CholS
Cer (*S*)-AS	Cer (*S*)-AS/FFAs/Chol (1:1:1 mol) + 5 wt% of CholS
Cer NdS	Cer NdS/FFAs/Chol (1:1:1 mol) + 5 wt% of CholS
Cer (*R*)-AdS	Cer (*R*)-AdS/FFAs/Chol (1:1:1 mol) + 5 wt% of CholS
Cer (*S*)-AdS	Cer (*S*)-AdS/FFAs/Chol (1:1:1 mol) + 5 wt% of CholS
Cer NP	Cer NP/FFAs/Chol (1:1:1 mol) + 5 wt% of CholS
Cer (*R*)-AP	Cer (*R*)-AP/FFAs/Chol (1:1:1 mol) + 5 wt% of CholS
Cer (*S*)-AP	Cer (*S*)-AP/FFAs/Chol (1:1:1 mol) + 5 wt% of CholS

**Table 2:** Main phase transition temperatures deduced from methylene symmetric stretching in Cer/(*d*)-FFAs/Chol/CholS membranes containing either sphingosine (Cer NS, Cer (*R*)-AS, or Cer (*S*)-AS), or dihydrosphingosine (Cer NdS, Cer (*R*)-AdS, or Cer (*S*)-AdS) or phytosphingosine Cers (Cer NP, Cer (*R*)-AP, or Cer (*S*)-AP) at ambient room humidity. The numbers in brackets indicate doublet disappearance (deduced from methylene scissoring vibration), which indicates an orthorhombic–hexagonal transition.

	Unlabeled Membranes	Membranes with *d*-FFA [°C]	
	[°C]	Cer Chains	*d*-FFA Chains
Cer NS	57 (37)	55	55
Cer (*R*)-AS	63 (63)	61	59
Cer (*S*)-AS	71 (69)	67	59
Cer NdS	73 (65)	83	59
Cer (*R*)-AdS	83 (65)	95	59
Cer (*S*)-AdS	79 (77)	89	63
Cer NP	81 (60)	89	58
Cer (*R*)-AP	79 (60)	79	59
Cer (*S*)-AP	75 (74)	71	69

## Data Availability

Data are contained within the article or supplementary material.

## Supplementary Materials

The following are available online at https://www.mdpi.com/article/10.3390/ijms22147468/s1.
