# Supplementary material for: Effects of (R)- and (S)-α-Hydroxylation of Acyl Chains in Sphingosine, Dihydrosphingosine, and Phytosphingosine Ceramides on Phase Behavior and Permeability of Skin Lipid Models
Source: Int J Mol Sci. 2021 Jul 12;22(14):7468. doi: 10.3390/ijms22147468 (PMC8303283; doi:10.3390/ijms22147468)
Supplement: Supplementary file 1 [file ijms-22-07468-s001.zip › Supplementary Material.pdf]

SUPPLEMENTARY MATERIAL

Effects of (*R*)- and (*S*)-α-Hydroxylation of Acyl Chain in Sphingosine, Dihydrosphingosine, and Phytosphingosine Ceramides on Phase Behavior and Permeability of Skin Lipid Models

Andrej Kováčik[a,*], Petra Pullmannová[a], Lukáš Opálka[a], Michaela Šilarová[a], Jaroslav Maixner[b], Kateřina Vávrová[a]

[a] *Skin Barrier Research Group, Faculty of Pharmacy in Hradec Králové, Charles University, Akademika Heyrovského 1203, 500 05 Hradec Králové, Czech Republic*

[b] *Faculty of Chemical Technology, University of Chemistry and Technology in Prague, Technická 5, 166 28 Prague, Czech Republic*

**Correspondence:* Andrej Kováčik, E-mail: kovacika@faf.cuni.cz

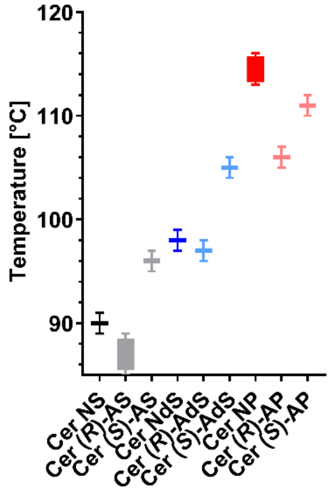

Figure S1. Melting temperatures of pure non-hydroxylated (Cer NS, Cer NdS, and Cer NP), α-hydroxylated Cer (Cer (*R*)-AS, Cer (*R*)-AdS, and Cer (*R*)-AP) and their non-physiological counterparts (Cer (*S*)-AS, Cer (*S*)-AdS, and Cer (*S*)-AP).

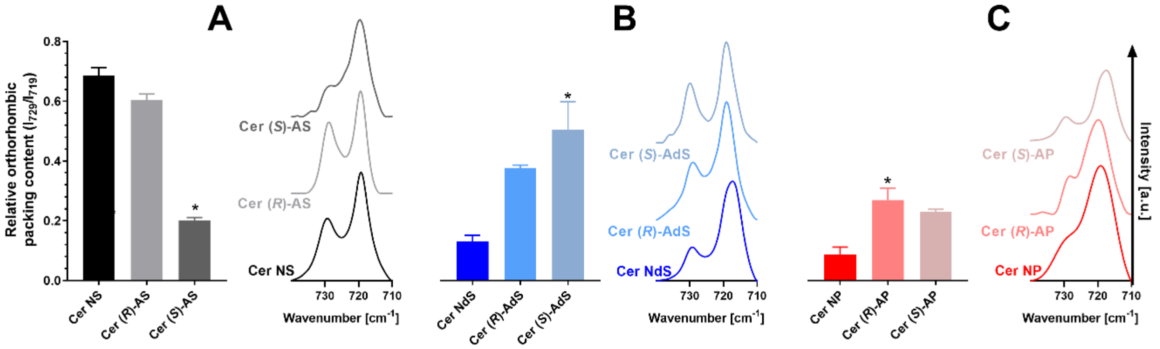

Figure S2. Lateral lipid packing in the model membranes containing the studied Cers: sphingosine Cers (panel A: Cer NS, Cer (*R*)-AS, and Cer (*S*)-AS), dihydrosphingosine Cers (panel B: Cer NdS, Cer (*R*)-AdS, and Cer (*S*)-AdS) and phytosphingosine Cers (panel C: Cer NP, Cer (*R*)-AP, and Cer (*S*)-AP), FFA, Chol, and CholS. The first graphs in each panel represent the relative content of the orthorhombic packing calculated from the methylene rocking contour at 32°C (shown in the second graphs); mean ± SEM. Asterisks indicate the statistical significance against the control (membrane with non-hydroxylated Cer).

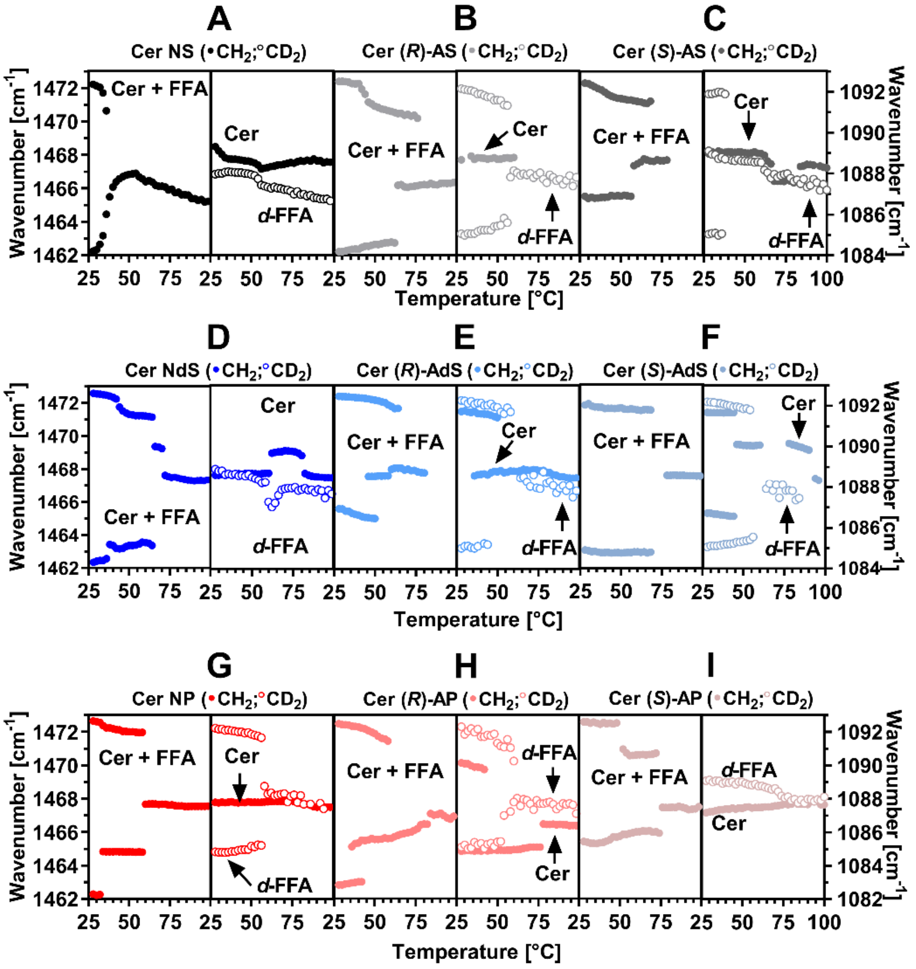

Figure S3. Thermotropic behavior of the major methylene scissoring bands of the model SC lipid membranes containing the studied Cers: sphingosine Cers (Cer NS, Cer (*R*)-AS, and Cer (*S*)-AS), dihydrosphingosine Cers (Cer NdS, Cer (*R*)-AdS, and Cer (*S*)-AdS) and phytosphingosine Cers (Cer NP, Cer (*R*)-AP, and Cer (*S*)-AP), (*d*)-FFAs, Chol, and CholS. Filled circles: CH_2 bands (unlabeled lipids or mainly Cer chains in samples with *d*-FFAs), open circles: CD_2 bands (*d*-FFAs). Two circles at a given temperature represent splitting the scissoring band into a doublet, which indicates an orthorhombic packing.
